# Investigation of Pharmacologically Important Polyphenolic Secondary Metabolites in Plant-based Food Samples Using HPLC-DAD

**DOI:** 10.3390/plants13101311

**Published:** 2024-05-10

**Authors:** Naheed Akhtar, Amna Jabbar Siddiqui, Muhammad Ramzan, Jalal Uddin, Mufarreh Asmari, Hesham R. El-Seedi, Syed Ghulam Musharraf

**Affiliations:** 1 H.E.J. Research Institute of Chemistry, International Center for Chemical and Biological Sciences, University of Karachi, Karachi 75270, Pakistan; naheedakhtar41@yahoo.com (N.A.); rafridi73@gmail.com (M.R.); 2 Dr. Panjwani Center for Molecular Medicine and Drug Research, International Center for Chemical and Biological Sciences, University of Karachi, Karachi 75270, Pakistan; amna.jabbar@iccs.edu; 3 Department of Pharmaceutical Chemistry, College of Pharmacy, King Khalid University, Abha 61421, Saudi Arabia; jalaluddinamin@gmail.com (J.U.); masmri@kku.edu.sa (M.A.); 4 Department of Chemistry, Faculty of Science, Islamic University of Madinah, Madinah 42351, Saudi Arabia; hesham.el-seedi@ilk.uu.se; 5 The Affiliated T.C.M Hospital of Southwest Medical University, Luzhou 646600, China

**Keywords:** polyphenols, flavonoids, phenolic acid, coumarin, stilbenoid, HPLC-DAD

## Abstract

Polyphenolic compounds are vital components of plants. However, their analysis is particularly difficult and challenging due to their similar chemical and structural properties. In this study, we developed a simple and reproducible HPLC-DAD protocol for determining nineteen pharmacologically important polyphenols in plant-based food samples, including fruits (apple, banana, grapefruit, peach, grapes, plum, and pear), vegetables (onion, cabbage, capsicum, garlic, lemon, tomato, potato, and spinach), and other edible items (corn, kidney beans, green tea, black tea, and turmeric). The reference standards were pooled into four different groups based on logP values and expected retention time to avoid compound co-elution. These developed methods will be useful for the qualitative and quantitative analysis of biologically important polyphenolic compounds in various food samples and botanicals.

## 1. Introduction

Polyphenols represent the most prevalent and abundant group of phytochemicals, encompassing phenolic acids, flavonoids tannins, and lignins. They can be broadly classified into two major groups: flavonoids and non-flavonoids [1]. Among these, flavonoids stand out as the most studied and diverse class of polyphenols, boasting an extensive range of structural diversity, ecological importance, and a variety of physiological and biological actions. With over 10,000 flavonoids reported since their discovery, they rank as the third most prevalent class in the plant kingdom [2]. The biological activity of flavonoids is dependent on their chemical structure and the various moieties that are present within the compounds [3]. Plant-based foods, including fruits (such as grapefruits, oranges, berries, bananas, peaches, red and black currants, pomegranates, pears, grapes, and apples), vegetables (such as onions, potatoes, parsley, cabbage, eggplants, broccoli, spinach, cauliflower, beans, tomatoes, and peppers), beverages (such as tea), pulses, and chocolates, serve as rich sources of flavonoids [4].

Polyphenolic compounds, including flavonols, flavones, flavanones, phenolic acids, coumarins, and stilbenoids, exhibit numerous pharmacological activities such as antioxidant, antifungal, antidiabetic, anti-inflammatory, antimicrobial, antiallergic [5], antinociceptive [6], anti-osteoporosis, antitumor [7], antilipolytic, antiulcer, antiasthmatic, anti-tubercular [8], antibacterial, antimutagenic, anticancer, metabolic enzyme-modulating [9], antidepressant, antiviral [10], anti-carcinogenic [11], antiobesity, cardiovascular protective, hepatoprotective [12], antifibrotic [13], and antiangiogenic activities [14].

Non-flavonoid polyphenols possess a basic structure consisting of a single aromatic ring and include phenolic acids, stilbenes, xanthones, lignans, and tannins. Phenolic acids, the primary group of non-flavonoid polyphenols, contain a carboxylic acid functional group and can be further classified into hydroxybenzoic acids (such as gallic acid, p-hydroxybenzoic acid, protocatechuic acid, syringic acid, and vanillic acid) and hydroxycinnamic acids (including caffeic acid, ferulic acid, p-coumaric acid, chlorogenic acid, and sinapic acids) [15] based on their C1–C6 and C3–C6 structures, respectively [16].

Both flavonoids and non-flavonoid polyphenols exert significant impacts on human health due to their remarkable benefits [17], leading to their widespread utilization in the functional food, nutraceutical [18], cosmetic, and pharmaceutical industries [19].

Among the various analytical techniques, high-performance liquid chromatography (HPLC) coupled with a diode array detector (DAD) stands out for its simplicity and effectiveness, making it the most popular technique for qualitatively and quantitatively investigating natural polyphenols in various botanical products [20]. Numerous analytical methods have been reported for analyzing complex plant extracts using HPLC combined with different detection systems, including UPLC-DAD [21], HPLC-DAD-MS [22], HPLC-ELSD [23], HPLC-PDA [24], HPLC-PDA-MS [25], and LC-MS [26].

The primary aim of this study was to develop a simple protocol for the analysis of nineteen prevalent plant-based polyphenols, including flavonols, flavanones, flavones, phenolic acids, coumarins, and stilbenoids, using HPLC-DAD. These bioactive metabolites are among the most common secondary metabolites and are found in a wide variety of plants, food samples, and their formulations (such as pharmaceuticals and nutraceuticals). The developed methods underwent testing and validation using selected food samples, including fruits, vegetables, and other edible items. These protocols will be valuable for quality control-testing laboratories that are involved in the analysis of plant-based herbal formulations and nutraceuticals.

## 2. Results and Discussion

### 2.1. HPLC-DAD Method Optimization

LogP serves as a measure of a molecule’s hydrophilicity or hydrophobicity, indicating its partition between an aqueous and organic phase. This property crucially influences the elution time and potential co-elution with other substances in liquid chromatography (LC) methods. In the present study, a pooling strategy for common polyphenols based on logP values was employed to develop efficient and rapid separation methods. This strategy involved grouping compounds with varying logP values into small clusters, resulting in improved separation and prevention of co-elution. The advantage of this pooling strategy over single-compound screening lies in its ability to significantly reduce the analysis time, workload, cost, sample volume requirements, and likelihood of compound co-elution. Compounds with diverse logP values were carefully selected and pooled to mitigate the presence of co-eluting metabolites within the same pool.

All compounds in the four different pools were eluted in less than eight minutes. The total analysis time for efficient compound separation in the four pools ranged from 8 to 10 min, with a post-run duration of 1 min. The flow rate was maintained at 0.35 mL/min, and the injection volume was set at 2 μL. The mobile phase gradient was individually optimized for each pool. Table 1 presents the optimized HPLC parameters, including the flow rate, injection volume, and mobile phase gradient, for pools 1–4.

Eight different wavelengths (205, 210, 251, 254, 260, 280, 300, and 360 nm) were initially selected for analysis. Among these, 300 nm was determined as the most suitable wavelength for all pools due to satisfactory peak intensities and a lower signal-to-noise ratio compared to the other wavelengths selected.

Pool-1, -3, and -4 each contained five compounds: rutin, myricitrin, apigenin, diosmetin, and galangin; isoquercitrin, myricetin, quercetin, chrysin, and kaempferide; and chlorogenic acid, trans-ferulic acid, trans-resveratrol, herniarin, and cinnamic acid, respectively. Pool-2 contained four compounds: hyperoside, hesperidin, naringenin, and kaempferol.

**Figure 1:**
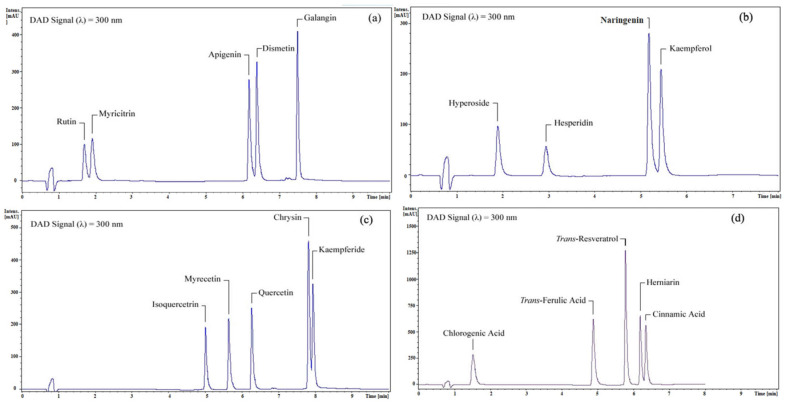
The optimized chromatogram of (**a**) Pool-1, (**b**) Pool-2, (**c**) Pool-3, and (**d**) Pool-4.

In Pool-1, rutin eluted at 1.7 min, myricitrin at 1.9 min, apigenin at 6.2 min, diosmetin at 6.4 min, and galangin at 7.5 min. In Pool-2, hyperoside eluted at 1.9 min, hesperidin at 2.9 min, naringenin at 5.2 min, and kaempferol at 5.4 min. In Pool-3, isoquercitrin eluted at 5.0 min, myricetin at 5.7 min, quercetin at 6.3 min, chrysin at 7.8 min, and kaempferide at 7.9 min. In Pool-4, chlorogenic acid eluted at 1.5 min, trans-ferulic acid at 4.9 min, trans-resveratrol at 5.8 min, herniarin at 6.2 min, and cinnamic acid at 6.3 min. The optimized chromatogram of each pool is depicted in Figure 1.

### 2.2. Method Performance and Validation

The validation of the developed method was conducted following international guidelines [27]. The optimized chromatographic conditions were utilized for analyzing seven different concentration levels in each pool, ranging from 50 to 1000 μg/mL. Calibration curves were constructed for each standard compound in their respective pools. The developed method exhibited excellent linearity, with high correlation coefficients (R^2^ = 0.9999–1) for each standard in all pools. The limits of detection (LODs) and limits of quantification (LOQs) ranged from 4.42 to 10.17 μg/mL and 13.38 to 30.83 μg/mL, respectively. Table 2 presents the regression equations, R-squared values, LODs, and LOQs of all compounds in their respective pools. The concentration of each compound was calculated using the regression equation.

The accuracy and precision of the developed method were assessed using bias and relative standard deviation (RSD). The precision and repeatability were evaluated for intra-day and inter-day analyses over three consecutive days. The triplicates of three different concentration levels (100, 400, and 800 μg/mL) of each pool were analyzed. The accuracy of the developed method for each pool was found to be greater than 95%, and the precision was found to be less than 5% in all cases. The %Accuracy and %RSD are presented in Supplementary Table S1.

To validate the developed method’s applicability, a recovery study was conducted by analyzing three selected spiked food samples at three different concentrations: 100 μg/mL (S1), 200 μg/mL (S2), and 300 μg/mL (S3). While most of the samples exhibited 95% recoveries using this approach, the combined analysis of all samples showed recoveries ranging from 89% to 106% (refer to Supplementary Table S2).

To assess the effectiveness of the developed methods, each pool was analyzed using every developed LC gradient method to verify the overlapping of retention times. Some compounds were observed to have the same retention time in various pools, such as myricitrin and hyperoside (Rt: 1.9 min), apigenin and herniarin (Rt: 6.2 min), and quercetin and cinnamic acid (Rt: 6.3 min). For better identification, compounds with similar retention times in each pool were analyzed using the other three methods developed. It was found that compounds in each pool exhibited a shift in retention times when analyzed using the other methods, resulting in a complete change in the profile of each pool compared to its respective developed method (refer to Supplementary Figure S1). This validation process proved to be instrumental to identifying the analyzed compounds in extracts of food samples.

### 2.3. Application of the Method on Plant Extracts

The optimized parameters were utilized to identify and quantify selected metabolites in various plant samples that are commonly used as food, including fruits, vegetables, and other edible items. The compound identification was based on retention times compared to standard compounds in each pool. The quantification results revealed analyte concentrations ranging from 20.7 to 2483.1 mg/kg in selected food samples.

Rutin was quantified in the range of 37.8–337.4 mg/kg, with abundant levels being found in plum (277.5 mg/kg), pear (124.9 mg/kg), spinach (166.1 mg/kg), green tea (337.4 mg/kg), and black tea (224.1 mg/kg). Myricitrin ranged from 75.4 to 398.2 mg/kg and was predominantly present in apple (228.0 mg/kg), onion (240.0 mg/kg), spinach (398.2 mg/kg), corn (148.2 mg/kg), and black tea (121.1 mg/kg). Apigenin was quantified in the range of 31.9–124.2 mg/kg and was mainly found in grapefruit (124.2 mg/kg), while diosmetin was quantified from 25.8 to 137.6 mg/kg and was only abundant in turmeric (137.6 mg/kg). Galangin ranged from 75.6 to 122.5 mg/kg, with higher levels being found in grapefruit (122.5 mg/kg) and turmeric (100.2 mg/kg).

Hyperoside was quantified from 44.9 to 1098.3 mg/kg and was abundant in apple (318.02 mg/kg), onion (332.8 mg/kg), green tea (1098.3 mg/kg), and black tea (273.9 mg/kg). Hesperidin ranged from 37.8 to 2483.2 mg/kg and was primarily found in banana (245.7 mg/kg), grapefruit (2483.2 mg/kg), lemon (303.2 mg/kg), and kidney beans (169.0 mg/kg). Naringenin was quantified from 39.3 to 108.9 mg/kg and was predominantly present in grapefruit (109.0 mg/kg), while kaempferol ranged from 30.9 to 69.3 mg/kg, with lower levels being observed in all samples.

Isoquercitrin ranged from 29.8 to 319.3 mg/kg and was abundant in apple (319.3 mg/kg), kidney beans (201.1 mg/kg), and green tea (227.7 mg/kg). Myricetin ranged from 37.5 to 301.7 mg/kg and was mainly found in apple (199.8 mg/kg), grapefruit (301.7 mg/kg), lemon (215.2 mg/kg), and green tea (182.2 mg/kg). Quercetin ranged from 31.0 to 190.3 mg/kg and was predominantly present in garlic (190.3 mg/kg) and lemon (172.3 mg/kg). Chrysin ranged from 33.0 to 112.7 mg/kg, with higher levels being observed in kidney beans (112.7 mg/kg), while kaempferide ranged from 48.9 to 118.3 mg/kg and was mainly abundant in turmeric (118.3 mg/kg).

Chlorogenic acid ranged from 34.7 to 216.4 mg/kg, with higher levels being found in apples (210 mg/kg), peach (200.6 mg/kg), and potato (153.2 mg/kg). Trans-ferulic acid ranged from 22.2 to 269.2 mg/kg and was predominantly present in grapefruit (157.0 mg/kg), spinach (142.5 mg/kg), corn (157.0 mg/kg), kidney beans (132.8 mg/kg), and green tea (269.2 mg/kg). Trans-resveratrol ranged from 38.2 to 145.1 mg/kg and was mainly abundant in grapefruit (80.3 mg/kg) and black tea (145.1 mg/kg) and below the limit of quantification (LOQ) in both corn and kidney beans. Herniarin was only present in grapefruit (36.0 mg/kg), while cinnamic acid ranged from 20.8 to 48.4 mg/kg and was less abundant in banana (48.4 mg/kg), grapes (23.0 mg/kg), plum (35.2 mg/kg), and corn (20.8 mg/kg).

Samples showing the presence of metabolites with similar retention times were analyzed against all the gradient systems used for Pools-1–4. Compounds such as myricitrin/hyperoside and apigenin/herniarin were detected in the same samples, with myricitrin/hyperoside being quantified in apple (240.0/332.8 mg/kg), onion (240.0/332.8 mg/kg), and black tea (121.1/273.9 mg/kg). Meanwhile, apigenin/herniarin were detected in grapefruit (124.2/36.0 mg/kg). The results indicated that the retention times of compounds shifted when using other methods compared to their respective developed method, enabling reliable detection of these compounds in the same food samples. Although quercetin and cinnamic acid exhibited similar retention times (6.3 min), they were not detected in the same samples.

**Figure 2:**
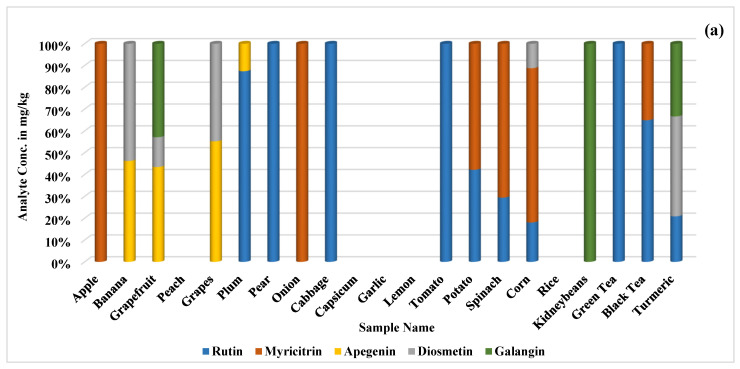

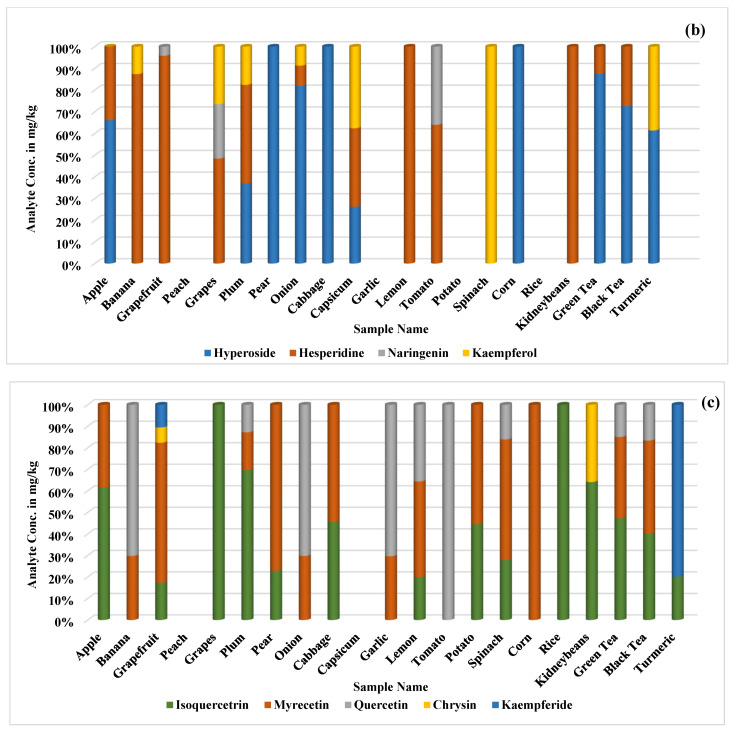

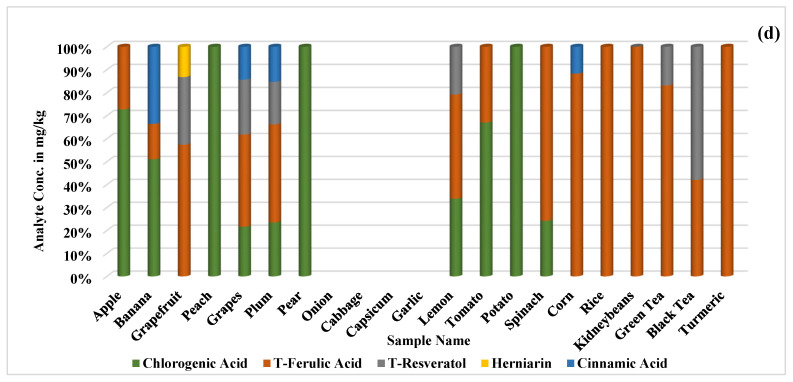
Visual representation of quantified polyphenolic compounds of (**a**) Pool-1, (**b**) Pool-2, (**c**) Pool-3, and (**d**) Pool-4 in food samples.

The quantified results of food samples in mg/kg are presented in Supplementary Table S3, while Figure 2 provides a visual representation of the quantified polyphenolic compounds of Pools-1–4 in food samples. Additionally, the comparison of standard compounds of each pool with the analyzed real samples is shown in Supplementary Figure S2.

### 2.4. Comparison with the Reported Methods

Several HPLC methods have been reported for the determination and quantification of different polyphenols. The current method is also compared with the previously reported methods. A detailed comparison of the current study with the reported methods is listed in Table 3.

In all reported methods, polyphenolic compounds were found to be highly retained in the column, resulting in longer retention times. Compared with previously reported studies, we observed that none of the mentioned methods address the rapid separation of the studied polyphenols within short time intervals. Our study achieves compound separation within a shorter time frame, leading to shorter elution times. Additionally, employing a pooling strategy facilitates efficient compound separation and mitigates the risk of co-elution.

The current study utilized calibration points ranging from 50 to 1000 μg/mL, with LOD and LOQ values ranging from 4.42 to 10.17 μg/mL and 13.38 to 30.83 μg/mL, respectively. However, the developed protocol demonstrates efficiency through a pooling strategy based on the log P values of nineteen polyphenols, categorized into four different pools. This method stands out for its simplicity, speed, and effectiveness in both sample preparation and analysis protocols.

## 3. Materials and Methods

### 3.1. Chemical and Reagents

**Figure 3:**
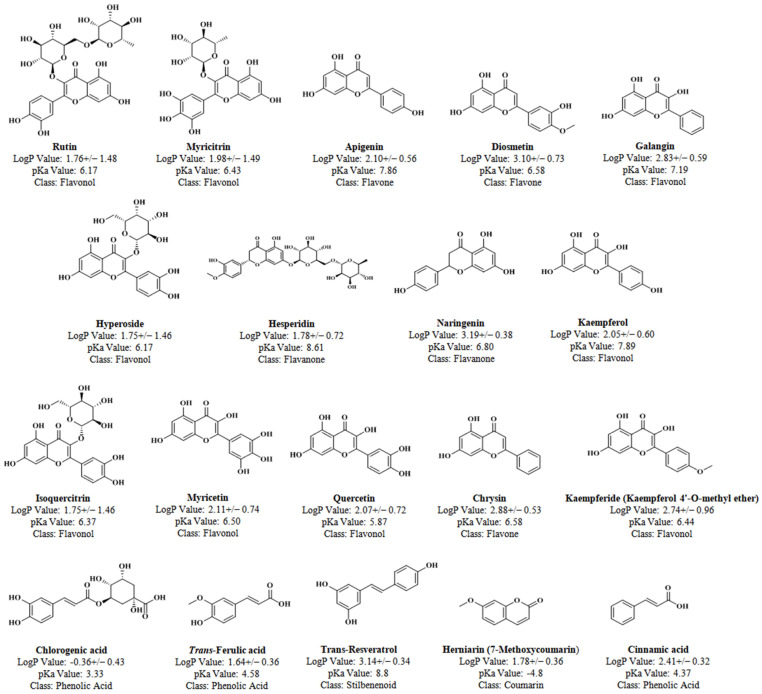
Names, classes, logP-values, and structures of studied compounds.

All solvents, chemicals, and standards utilized in this study were of HPLC grade. Formic acid, procured from Daejung Chemicals & Metals Co. Ltd., Incheon, Republic of Korea, served as an additive for the mobile phase. Methanol (MeOH) and acetonitrile (ACN) for the mobile phase were acquired from Merck KGaA, Darmstadt, Germany, and Daejung Chemicals & Metals Co. Ltd., Korea, respectively. Type-1 water obtained from the Ultrapure Water Purification assembly (Branstead^TM^ GenPure^TM^, Waltham, MA, USA), was utilized as the mobile phase throughout the study. Nineteen standard compounds were procured from Sigma Aldrich, USA. The names, classes, logP values, and structures of the standard compounds are depicted in Figure 3.

PubChem CIDs of studied compounds:

Rutin (PubChem CID: 5280805); Myricitrin (PubChem CID: 5281673); Apigenin (PubChem CID: 5280443); Diosmetin (PubChem CID: 5281612); Galangin (PubChem CID: 5281616); Hyperoside (PubChem CID: 5281643); Hesperidin (PubChem CID: 10621); Naringenin (PubChem CID: 932); Kaempferol (PubChem CID: 5280863); Isoquercitrin (PubChem CID: 5280804); Myricetin (PubChem CID: 5281672); Quercetin (PubChem CID: 5280343); Chrysin (PubChem CID: 5281607); Kaempferide (PubChem CID: 5281666); Chlorogenic acid (PubChem CID: 1794427); *Trans*-ferulic acid (PubChem CID: 445858); *Trans*-resveratrol (PubChem CID: 445154); Herniarin (PubChem CID: 10748); Cinnamic acid (PubChem CID: 444539).

### 3.2. Preparation of Standard Solution

Each standard compound was dissolved in 1 mL of methanol to prepare standard stock solutions at a concentration of 1 mg/mL. All nineteen compounds were then divided into four separate pools based on their logP values and expected retention times to prevent compound co-elution. Pool-1, Pool-3, and Pool-4 consisted of 5 compounds each, while Pool-2 contained 4 compounds. Each pool was created by mixing the respective standards to achieve a final concentration of 1 mg/mL in methanol for subsequent HPLC analysis. Calibration curves for each standard pool were constructed using seven calibrators ranging from 50 to 1000 μg/mL, prepared via the serial dilution method. Additionally, a sample from the standard stock solution was diluted fivefold (200 μL standard stock solution + 800 μL methanol) to optimize the chromatogram.

### 3.3. Sample Collection

Twenty-one polyphenol-rich food samples, comprising fruits (apple, banana, grapefruit, peach, grapes, plum, and pear), vegetables (onion, cabbage, capsicum, garlic, lemon, tomato, potato, and spinach), and other edible items (corn, kidney beans, green tea, black tea, and turmeric), were procured from supermarkets. The peel and pulp of fruits and vegetables were utilized for sample preparation. All food samples underwent thorough cleaning and were subsequently immersed in liquid nitrogen, crushed, and homogenized using a mortar. The resulting powder was then stored at −20 °C until extraction and analysis.

### 3.4. Sample Extraction and Preparation

All samples were prepared using a modified quick extraction method. Pre-homogenized and powdered food samples (5 ± 0.3 g) were mixed with 5 mL of extraction solvent (*v*/*v*) comprising methanol/water/formic acid (80:19:1). The samples underwent sonication for 24 h at room temperature, followed by centrifugation at 10,000 rpm for 10–15 min. The resulting supernatant was collected and filtered through PTFE syringe filters (0.22 μm) before being stored at −20 °C for subsequent HPLC analysis. The concentrations of detected and quantified compounds were calculated in μg/g.

### 3.5. HPLC-DAD Analysis

The chromatographic separation was conducted using an Agilent Technologies 1260 series HPLC system (Waldbronn, Germany), equipped with an auto-sampler, column thermostat column compartment, degasser, and a binary pump coupled with a DAD. Data processing was carried out using Agilent (1260) 2D ChemStation software Rev. B.04.02. To separate analytes in pools (1, 2, 3, and 4), various chromatographic parameters such as mobile phase gradient, flow rate, and injection volume were optimized individually to achieve well-separated peaks of the targeted analytes. The optimization of different HPLC parameters was performed using a trial-and-error method, with the tested parameters listed in Supplementary Table S4 for the final optimization of HPLC methods.

The separation was conducted on a reverse-phase Agilent SB-C18 column (3 × 50 mm, 1.8 μm) with a constant temperature of 30 °C set for the column compartment thermostat. The mobile phase consisted of 0.1% formic acid with deionized water as solvent A and acetonitrile (ACN) as solvent B, resulting in well-separated peaks of the analytes of interest. Both solvents were sonicated for 15 min prior to analysis. HPLC analysis parameters, including flow rate, injection volume, and mobile phase gradient, are provided in Table 1. To ensure proper equilibration of the column environment, a post-run equivalent to one minute was added at both ends of the gradient program.

For analysis, wavelengths of 205, 210, 251, 254, 260, 280, 300, and 360 nm were selected. All standard and food samples, including fruits, vegetables, and others, were analyzed in triplicate according to the developed method using the HPLC-DAD system. Compounds in food samples were identified based on common factors such as retention times and UV spectra at selected wavelengths.

### 3.6. Method Validation

The method validation encompassed the assessment of various analytical parameters, including calibration, linearity, accuracy, precision, limit of detection (LOD), limit of quantification (LOQ), and recovery studies, following international validation protocols [27]. For quantification purposes, the linearity of the developed method was confirmed through calibration curves that were generated using eight different concentration levels of all pools, prepared by appropriately diluting standard stock solutions. The linear calibration curves were constructed by analyzing seven calibration points ranging from 50 to 1000 μg/mL in triplicate. Accuracy and precision were evaluated in terms of %Bias and %RSD, respectively. Intra-day and inter-day analyses were conducted by analyzing three replicates at three different known concentrations on a single day and over three consecutive days. The LOD and LOQ were determined using the formulas 3.3 σ/S and 10 σ/S, respectively, where σ represents the standard deviation of the response and S represents the slope of the calibration curve. The accuracy of the developed method was assessed through recovery studies. Recovery analysis involved spiking original samples at three different fortified concentrations of standards (S1, S2, and S3 μg/mL) in each pool. Results for recovery studies were reported as percentage recoveries.

## 4. Conclusions

In this study, a straightforward protocol employing a pooling strategy was developed for the investigation and quantification of nineteen prevalent polyphenols across diverse food samples using HPLC-DAD. Utilizing optimized conditions, the proposed method yielded statistically satisfactory outcomes in terms of linearity, accuracy, precision, LOD, and LOQ. The pooling strategy, based on the logP values of reference standards, renders this method an efficient approach for the swift determination of a range of phenolic compounds in different food samples. Additionally, the developed method holds promise for rapid profiling of selected polyphenols in processed food samples and nutraceuticals.

## Figures and Tables

**Table 1:** Optimized HPLC parameters, flow rates, injection volumes, and mobile phase gradients for Pools-1–4.

Pool No.	Flow Rate (mL/min)	Gradient	Compound Name (RT)
POOL–1	0.35 mL/min	20–30% B, 0–1 min; 30–40% B, 1–3 min; 40–80% B, 3–5 min; 80–40% B, 5–7 min; 40–30% B, 7–8 min; 30–20% B, 8–10 min	Rutin (1.7), myricitrin (1.9), apigenin (6.2), diosmetin (6.4), and galangin (7.5)
POOL–2	0.35 mL/min, (0–3) min, 0.5 mL/min, (4–5) min, 0.35 mL/min, (5–8) min	20–30% B, 0–1 min; 30–40% B, 1–3 min; 40–70% B, 3–4.5 min; 70–40% B, 4.5–6.5 min; 40–30% B, 6.5–7 min; 30–20% B, 7–8 min	Hyperoside (1.9), hesperidin (2.9), naringenin (5.2), and kaempferol (5.4)
POOL–3	0.35 mL/min	10–20% B, 0–1 min; 20–70% B, 1–5 min; 70% B, 5–7.5 min; 70–20% B, 7.5–8 min; 20–10% B, 8–10 min	Isoquercitrin (5.0), myricetin (5.7), quercetin (6.3), chrysin (7.8), and kaempferide (7.9)
POOL–4	0.35 mL/min	10% B, 0–1 min; 10–100% B, 1–6 min; 100–10% B, 6–7 min; 10% B, 7–8 min	chlorogenic acid (1.5), *trans*-ferulic acid (4.9), *trans*-resveratrol (5.8), herniarin (6.2), and cinnamic acid (6.3)

Column: Agilent SB C-18; column dimensions: 3 × 50 mm, 1.8 μm. Injection volume: 2 μL; temperature: 30 °C.

**Table 2:** Regression equation, R-squared values, LODs, and LOQs of all compounds.

Compound Name	Regression Equation	R^2^	LOD	LOQ
			μg/mL	μg/mL
Rutin	y = 2.7898x − 26.326	0.9999	5.92	17.94
Myricitrin	y = 3.9548x − 9.5223	0.9998	10.17	30.83
Apigenin	y = 6.3609x − 0.3116	0.9999	7.17	21.74
Diosmetin	y = 7.2119x + 1.6471	0.9999	6.64	20.13
Galangin	y = 7.0972x − 6.4245	1	5.82	17.63
Hyperoside	y = 3.0497x − 15.326	0.9999	5.91	17.90
Hesperidin	y = 2.0851x − 13.794	0.9999	6.04	18.31
Naringenin	y = 7.3752x − 53.448	1	4.42	13.38
Kaempferol	y = 5.9455x + 13.596	0.9999	6.35	19.24
Isoquercitrin	y = 3.8363x − 1.0036	0.9999	5.87	17.80
Myricetin	y = 4.768x − 9.6312	0.9999	6.42	19.46
Quercetin	y = 5.2848x − 57.539	1	4.49	13.60
Chrysin	y = 8.5065x + 10.403	0.9999	5.95	18.02
Kaempferide	y = 7.1759x − 133.44	0.9999	6.63	20.10
Chlorogenic Acid	y = 10.947x − 147.12	0.9999	6.28	19.02
*trans*-Ferulic Acid	y = 15.369x + 39.294	0.9999	5.92	17.95
*trans*-Resveratrol	y = 19.61x + 116.61	0.9999	5.88	17.81
Herniarin	y = 11.892x − 143.54	0.9999	6.10	18.50
Cinnamic Acid	y = 11.24x − 15.634	0.9999	5.87	17.80

LOD: limit of detection; LOQ: limit of quantification.

**Table 3:** Comparison of the current study with the reported methods.

S. No.	Studied Analytes	Total Run Time of Analysis (min)	Flow Rate (mL/min)	LOD μg/mL	LOQ μg/mL	Ref.
Method-1	Kaempferol	85	0.8	-	-	[28]
	Quercetin			-	-	
	Chlorogenic acid			-	-	
	Ferulic acid			-	-	
	Resveratrol			-	-	
Method-2	Rutin	40	1.6	0.03742	0.11341	[20]
	Kaempferol			0.03621	0.10975	
	Myricetin			0.04477	0.13568	
	Quercetin			0.0154	0.04668	
Method-3	Rutin	60	0.6	0.028	0.096	[29]
	Kaempferol			0.021	0.069	
	Quercetin			0.019	0.063	
Method-4	Hyperoside	71	250	0.5	-	[21]
Method-5	Rutin	30	0.6	0.71	2.368	[30]
Method-6	Rutin	45	-	0.014	0.047	[31]
	Quercetin			0.004	0.013	
	Chrysin			0.004	0.014	
	Chlorogenic acid			0.006	0.02	
	Ferulic acid			0.001	0.03	
	Cinnamic acid			0.005	0.016	
Method-7	Myricetin	45	0.7	1.96	6.54	[32]
	Quercetin			2.88	9.59	
	Ferulic acid			0.16	0.21	
	Resveratrol			0.07	0.03	
	Cinnamic acid			0.03	0.04	
Current study	Rutin	8 to 10	0.35	5.92	17.94	-
	Myricitrin			10.17	30.83	
	Apigenin			7.17	21.74	
	Diosmetin			6.64	20.13	
	Galangin			5.82	17.63	
	Hyperoside			5.91	17.9	
	Hesperidin			6.04	18.31	
	Naringenin			4.42	13.38	
	Kaempferol			6.35	19.24	
	Isoquercitrin			5.87	17.8	
	Myricetin			6.42	19.46	
	Quercetin			4.49	13.6	
	Chrysin			5.95	18.02	
	Kaempferide			6.63	20.1	
	Chlorogenic Acid			6.28	19.02	
	*Trans*-Ferulic Acid			5.92	17.95	
	*Trans*-Resveratrol			5.88	17.81	
	Herniarin			6.1	18.5	

LOD: limit of detection; LOQ: limit of quantification; -: not mentioned.

## Data Availability

Dataset available on request from the authors.

## Supplementary Materials

The following supporting information can be downloaded at https://www.mdpi.com/article/10.3390/plants13101311/s1, Figure S1: The comparison of Pool-1–4 on different gradient systems.; Figure S2: The comparison of standard compounds of each pool ((a) Pool-1, (b) Pool-2, (c) Pool-3, and (d) Pool-4) with analyzed real samples. Table S1: The %accuracy and %RSD of all compounds; Table S2: Results of the percent recovery study; Table S3: The quantified results of food samples in mg/kg; Table S4: Tested parameters for the final optimization of HPLC method.
