# Supplementary material for: Investigation of Pharmacologically Important Polyphenolic Secondary Metabolites in Plant-based Food Samples Using HPLC-DAD
Source: Plants (Basel). 2024 May 10;13(10):1311. doi: 10.3390/plants13101311 (PMC11124887; doi:10.3390/plants13101311)
Supplement: Supplementary file 1 [file plants-13-01311-s001.zip › plants-2915513-supplementary.pdf]

Investigation of Pharmacologically Important Polyphenolic Secondary Metabolites in Plant-based Food Samples Using HPLC-DAD

Naheed Akhtar [1], Amna Jabbar Siddiqui [2], Muhammad Ramzan [1], Jalal Uddin [3], Mufarreh Asmari [3], Hesham R. El-Seedi [4] and Syed Ghulam Musharraf [1,2,5,*]

[1] H.E.J. Research Institute of Chemistry, International Center for Chemical and Biological Sciences, University of Karachi, Karachi 75270, Pakistan; naheedakhtar41@yahoo.com (N.A.); rafridi73@gmail.com (M.R.)
[2] Dr. Panjwani Center for Molecular Medicine and Drug Research, International Center for Chemical and Biological Sciences, University of Karachi, Karachi 75270, Pakistan; amna.jabbar@iccs.edu
[3] Department of Pharmaceutical Chemistry, College of Pharmacy, King Khalid University, Abha 61421, Saudi Arabia; jalaluddinamin@gmail.com (J.U.); masmri@kku.edu.sa (M.A.)
[4] Department of Chemistry, Faculty of Science, Islamic University of Madinah, Madinah, 42351, Saudi Arabia ; hesham.el-seedi@ilk.uu.se
[5] The Affiliated T.C.M Hospital of Southwest Medical University, Luzhou, 646600, China
* Correspondence: musharraf@iccs.edu or musharraf1977@yahoo.com; Tel.: +92-213-4824924-5; Fax: + 92-213-4819018-9

Supplementary Material

List of Supplementary Figure

Supplementary Fig. 1. The comparison of pools 1-4 on different gradient systems.

Supplementary Fig. 2. The comparison of standard compounds of each pool (a) pool-1, (b) pool-2, (c) pool-3, (d) pool-4 with analyzed real samples.

List of Supplementary Tables

Supplementary Table S1. The %accuracy and %RSD of all compounds.

Supplementary Table S2. The quantified results of food samples in mg/Kg.

Supplementary Table S3. Results of the percent recovery study.

Supplementary Table S4. Tested gradient systems for the final optimization of HPLC methods.

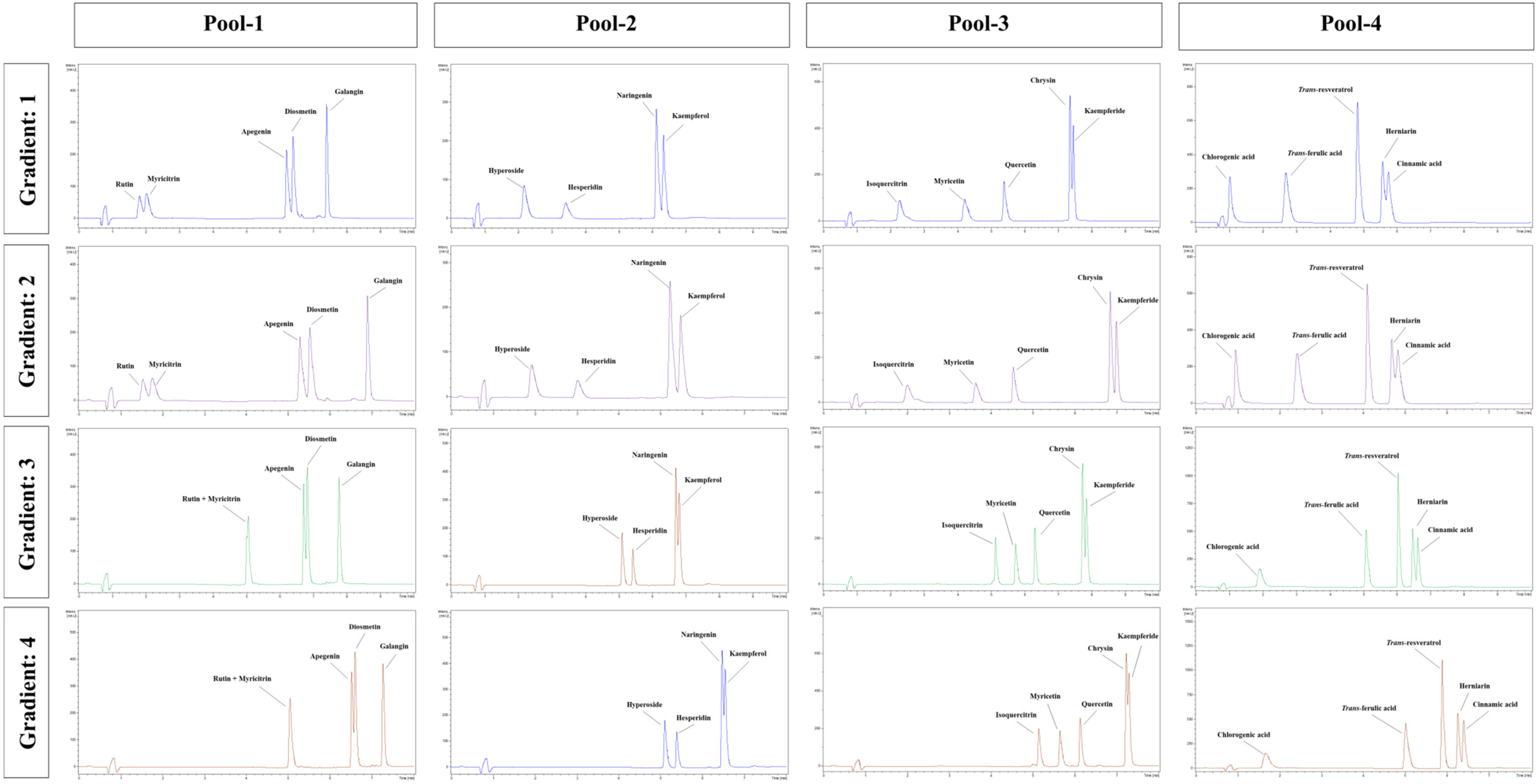

Pool-1: Rutin, Myricitrin, Apigenin, Diosmetin and Galangin
Pool-2: Hyperoside, Hesperidin, Naringenin and Kaempferol
Pool-3: Isoquercitrin, Myricetin, Quercetin, Chrysin and Kaempferide
Pool-4: Chlorogenic acid, *Trans*-ferulic acid, *Trans*-resveratrol, Herniarin and Cinnamic acid

Supplementary Fig. 1. The comparison of pools 1-4 on different gradient systems.

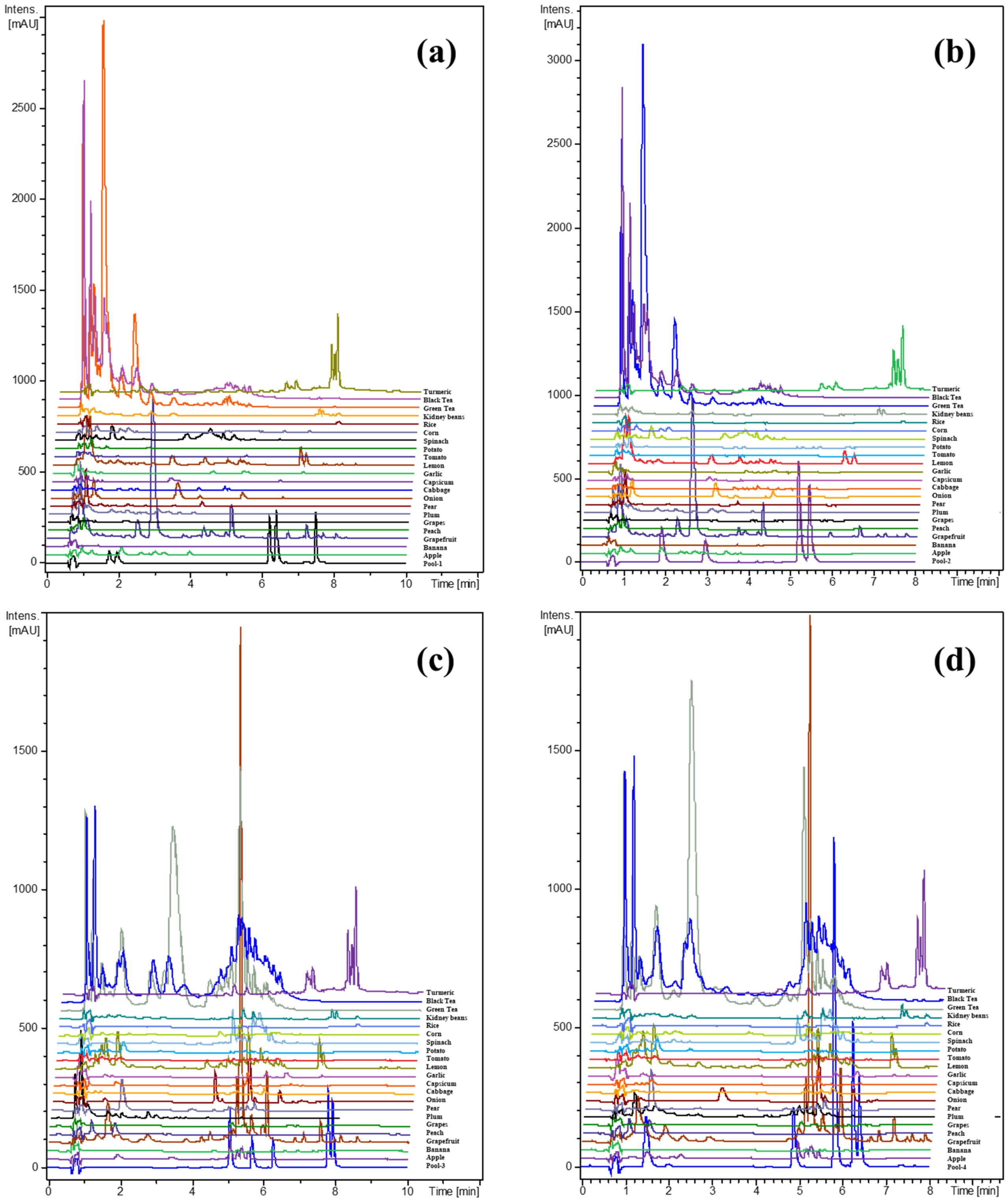

Supplementary Fig. 2. The comparison of standard compounds of each pool (a) pool-1, (b) pool-2, (c) pool-3, (d) pool-4 with analyzed real samples.

Supplementary Table S1. The %accuracy and %RSD of all compounds.

POOL-1							
**Compound Name**	**Concentrations in µg/ml**	**Intra-day**	**%RSD**	**%Accuracy**	**Inter-day**	**%RSD**	**%Accuracy**
		**Found concentration in µg/mL**			**Found concentration in µg/mL**		
**Rutin**	100	99.2 ± 0.9	0.91	99.21	96 ± 3	3.59	95.93
	400	399 ± 5	1.24	99.57	401 ± 3	0.69	100.15
	800	805 ± 5	0.60	100.60	806 ± 5	0.58	100.78
**Myricitrin**	100	103 ± 3	3.04	103.17	107 ± 2	1.56	107.26
	400	402.2 ± 0.2	0.04	100.53	405 ± 5	1.10	101.37
	800	793 ± 1	0.15	99.09	790 ± 2	0.21	98.77
**Apigenin**	100	102.5 ± 0.2	0.15	102.53	105 ± 4	4.01	105.54
	400	399 ± 2	0.60	99.63	401 ± 4	0.97	100.18
	800	801 ± 8	1.02	100.09	796 ± 7	0.86	99.50
**Diosmetin**	100	101.5 ± 0.2	0.21	101.47	104 ± 4	3.85	104.26
	400	402 ± 3	0.70	100.42	403 ± 2	0.50	100.69
	800	800 ± 3	0.40	99.97	796 ± 3	0.42	99.56
**Galangin**	100	99 ± 2	2.10	99.30	100 ± 2	2.01	99.59
	400	400 ± 1	0.37	100.06	402 ± 4	0.98	100.45

	800	797 ± 4	0.51	99.65	798 ± 1	0.16	99.79
**POOL-2**							
**Compound Name**	**Concentrations in µg/ml**	**Intra-day Found concentration in µg/mL**	**%RSD**	**%Accuracy**	**Inter-day Found concentration in µg/mL**	**%RSD**	**%Accuracy**
**Hyperoside**	100	103 ± 5	4.34	103.03	101 ± 4	3.92	101.39
	400	396 ± 1	0.25	99.02	412 ± 3	0.62	102.90
	800	798 ± 1	0.18	99.70	798 ± 2	0.23	99.74
**Hesperidin**	100	104 ± 3	2.67	104.14	103 ± 3	2.53	103.43
	400	400 ± 2	0.41	99.92	403.0 ± 0.4	0.09	100.76
	800	800 ± 2	0.27	100.06	808 ± 9	1.16	100.95
**Naringenin**	100	102 ± 1	1.41	101.56	102 ± 4	3.55	102.84
	400	400 ± 4	0.92	99.74	408 ± 1	0.23	102.01
	800	803± 4	0.45	100.43	804 ± 5	0.67	100.55
**Kaempferol**	100	98 ± 3	2.61	97.55	101 ± 3	2.80	100.62
	400	398 ± 3	0.73	99.59	399.3 ± 0.9	0.22	99.84
	800	805 ± 3	0.36	100.58	801 ± 3	0.34	100.17
**POOL-3**							
**Compound Name**	**Concentrations in**	**Intra-day Found**	**%RSD**	**%Accuracy**	**Inter-day Found**	**%RSD**	**%Accuracy**

	µg/ml	concentration in µg/mL			concentration in µg/mL		
**Isoquercitrin**	100	100 ± 2	2.29	99.83	101 ± 5	4.66	100.73
	400	402 ± 2	0.43	100.52	409 ± 8	1.90	102.27
	800	804 ± 7	0.85	100.50	820 ± 10	1.48	102.54
**Myricetin**	100	100 ± 1	1.16	100.24	104 ± 5	4.68	103.87
	400	406 ± 5	1.18	101.41	408.5 ± 0.2	0.06	102.13
	800	799 ± 3	0.40	99.91	795 ± 7	0.88	99.48
**Quercetin**	100	101 ± 1	1.24	100.89	104 ± 4	4.37	104.11
	400	397 ± 2	0.43	99.19	395 ± 2	0.50	98.88
	800	800 ± 2	0.23	99.98	799 ± 4	0.46	99.87
**Chrysin**	100	100 ± 1	1.22	99.56	99 ± 3	2.67	98.76
	400	398.2 ± 0.8	0.20	99.54	392 ± 8	1.91	97.99
	800	804 ± 1	0.13	100.46	807 ± 5	0.59	100.88
**Kaempferide**	100	103 ± 1	0.98	103.21	107 ± 4	4.04	107.12
	400	395 ± 2	0.42	98.67	398 ± 7	1.66	99.51
	800	800 ± 1	0.16	100.02	808 ± 10	1.20	101.02
**POOL-4**							
**Compound Name**	**Concentrations in µg/ml**	**Intra-day**	**%RSD**	**%Accuracy**	**Inter-day**	**%RSD**	**%Accuracy**
		**Found concentration in**			**Found concentration in**		

		µg/mL			µg/mL		
**Chlorogenic Acid**	100	101.6 ± 0.5	0.55	101.58	102 ± 2	2.02	102.41
	400	397.1 ± 0.8	0.20	99.28	394 ± 4	1.07	98.42
	800	805 ± 2	0.23	100.59	810 ± 5	0.65	101.29
***Trans*-Ferulic Acid**	100	103 ± 2	1.71	103.39	104 ± 2	2.03	103.55
	400	401 ± 1	0.36	100.16	393 ± 9	2.41	98.33
	800	798 ± 5	0.64	99.75	795 ± 5	0.66	99.32
***Trans*-Resveratrol**	100	98 ± 1	1.05	98.45	103 ± 4	4.16	102.60
	400	400 ± 2	0.47	100.09	400 ± 3	0.81	100.12
	800	800 ± 5	0.58	100.07	798 ± 4	0.47	99.75
**Herniarin**	100	102 ± 2	2.17	101.87	102 ± 2	2.07	101.80
	400	397 ± 2	0.64	99.12	397 ± 1	0.38	99.29
	800	802 ± 1	0.15	100.25	801 ± 3	0.44	100.09
**Cinnamic Acid**	100	99 ± 0.8	0.90	98.77	100 ± 2	2.21	100.14
	400	403 ± 3	0.86	100.72	402 ± 3	0.79	100.52
	800	804 ± 2	0.29	100.50	801.2 ± 0.6	0.07	100.16

Supplementary Table S2. shows the Results of the percent recovery study.

POOL-1							
**Sample Category**	**Sample Name**	**Spiking Level**	**% Recovery**				
			**Rutin**	**Myricitrin**	**Apigenin**	**Diosmetin**	**Galangin**
**Fruit**	**Grapefruit**	**S1**	94.18	102.94	96.34	96.12	92.80
		**S2**	98.16	98.23	92.78	93.17	106.59
		**S3**	104.54	101.69	95.11	97.04	103.01
**Vegetable**	**Spinach**	**S1**	92.98	97.59	96.42	101.52	95.42
		**S2**	98.55	106.96	104.66	98.06	93.69
		**S3**	96.61	103.58	102.62	91.51	91.66
**Other**	**Corn**	**S1**	94.18	102.94	96.34	96.12	92.80
		**S2**	98.16	98.23	92.78	93.17	106.59
		**S3**	104.54	101.69	95.11	97.04	103.01

POOL-2						
**Sample Category**	**Sample Name**	**Spiking Level**	**% Recovery**			
			**Hyperoside**	**Hesperidin**	**Naringenin**	**Kaempferol**
**Fruit**	**Grapefruit**	**S1**	93.48	91.01	105.35	101.64
		**S2**	103.69	102.96	98.11	93.48
		**S3**	97.68	97.68	101.60	103.46
**Vegetable**	**Spinach**	**S1**	104.21	96.01	87.34	98.38
		**S2**	97.43	104.92	93.00	93.83

		**S3**	96.11	95.53	90.57	95.38	
**Other**	**Corn**	**S1**	93.48	91.01	105.35	101.64	
		**S2**	103.69	102.96	98.11	93.48	
		**S3**	97.68	97.68	101.60	103.46	
**POOL-3**							
**Sample Category**	**Sample Name**	**Spiking Level**	**% Recovery**				
			**Isoquercitrin**	**Myricetin**	**Quercetin**	**Chrysin**	**Kaempferide**
**Fruit**	**Grapefruit**	**S1**	97.51	100.02	106.64	94.54	103.41
		**S2**	106.88	98.24	98.20	97.94	97.16
		**S3**	96.70	103.85	97.00	90.70	100.28
**Vegetable**	**Spinach**	**S1**	103.51	101.82	95.76	92.22	95.31
		**S2**	92.30	99.56	100.41	105.40	103.67
		**S3**	97.34	97.09	91.35	91.86	97.72
**Other**	**Corn**	**S1**	97.51	100.02	106.64	94.54	103.41
		**S2**	106.88	98.24	98.20	97.94	97.16
		**S3**	96.70	103.85	97.00	90.70	100.28
**POOL-4**							
**Sample Category**	**Sample Name**	**Spiking Level**	**% Recovery**				
			**Chlorogenic Acid**	***Trans*-Ferulic Acid**	***Trans*-Resveratrol**	**Herniarin**	**Cinnamic Acid**
**Fruit**	**Grapefruit**	**S1**	101.29	97.66	96.68	94.67	97.39

		**S2**	98.63	96.63	103.61	99.12	94.97
		**S3**	103.30	101.12	98.45	97.50	91.62
**Vegetable**	**Spinach**	**S1**	99.64	90.20	101.56	89.84	101.47
		**S2**	91.49	103.18	98.18	91.62	99.28
		**S3**	92.24	92.87	95.72	98.22	89.83
**Other**	**Corn**	**S1**	101.29	97.66	96.68	94.67	97.39
		**S2**	98.63	96.63	103.61	99.12	94.97
		**S3**	103.30	101.12	98.45	97.50	91.62

Supplementary Table S3. The quantified results of food samples in mg/Kg.

POOL-1						
**Analyte Conc. mg/kg of Food Samples**						
**Sample Category**	**Sample Name**	**Rutin**	**Myricitrin**	**Apigenin**	**Diosmetin**	**Galangin**
**Fruits**	**Apple**	ND	228.02 ± 1.5	ND	ND	ND
	**Banana**	ND	ND	63.19 ± 5.5	73.48 ± 1.5	ND
	**Grapefruit**	ND	ND	124.22 ± 0.9	39.00 ± 0.4	122.46 ± 0.8
	**Peach**	ND	ND	ND	ND	ND
	**Grapes**	ND	ND	31.87 ± 0.4	25.81 ± 1.3	ND
	**Plum**	277.45 ± 3.6	ND	40.04 ± 1.4	ND	ND
	**Pear**	124.89 ± 5.3	ND	ND	ND	ND
**Vegetables**	**Onion**	ND	240.02 ± 1.1	ND	ND	ND
	**Cabbage**	49.62 ± 1.8	ND	ND	ND	ND
	**Capsicum**	ND	ND	ND	ND	ND
	**Garlic**	ND	ND	ND	ND	ND
	**Lemon**	ND	ND	ND	ND	ND
	**Tomato**	93.36 ± 3.6	ND	ND	ND	ND
	**Potato**	55.00 ± 3.3	75.36 ± 0.5	ND	ND	ND
	**Spinach**	166.07 ± 0.4	398.18 ± 5.0	ND	ND	ND
**Others**	**Corn**	37.82 ± 0.7	148.24 ± 1.0	ND	23.51 ± 1.0	ND

	**Rice**	ND	ND	ND	ND	ND
	**Kidney beans**	ND	ND	ND	ND	75.56 ± 0.6
	**Green Tea**	337.35 ± 0.2	ND	ND	ND	ND
	**Black Tea**	224.12 ± 0.2	121.08 ± 0.6	ND	ND	ND
	**Turmeric**	62.69 ± 2.1	ND	ND	137.59 ± 1.3	100.20 ± 0.1

**POOL-2**					
**Analyte Conc. mg/kg of Food Samples**					
**Sample Category**	**Sample Name**	**Hyperoside**	**Hesperidin**	**Naringenin**	**Kaempferol**
**Fruits**	**Apple**	318.02 ± 2.3	162.62 ± 0.4	ND	ND
	**Banana**	ND	245.66 ± 0.9	ND	35.64 ± 0.5
	**Grapefruit**	ND	2483.16 ± 0.5	108.92 ± 2.0	ND
	**Peach**	ND	ND	ND	ND
	**Grapes**	ND	102.32 ± 1.4	52.77 ± 1.7	55.95 ± 1.2
	**Plum**	134.23 ± 1.6	165.16 ± 0.5	ND	63.94 ± 1.7
	**Pear**	110.28 ± 2.1	ND	ND	ND
**Vegetables**	**Onion**	332.78 ± 1.5	37.82 ± 0.9	ND	35.64 ± 5.8
	**Cabbage**	118.85 ± 1.4	ND	ND	ND
	**Capsicum**	44.93 ± 4.1	62.25 ± 0.4	ND	64.53 ± 1.0
	**Garlic**	ND	ND	ND	ND
	**Lemon**	ND	303.18 ± 3.1	ND	ND

	**Tomato**	ND	69.98 ± 4.8	39.34 ± 2.8	ND
	**Potato**	ND	ND	ND	ND
	**Spinach**	ND	ND	ND	69.30 ± 2.0
**Others**	**Corn**	45.57 ± 0.2	ND	ND	ND
	**Rice**	ND	ND	ND	ND
	**Kidney beans**	ND	168.96 ± 2.7	ND	ND
	**Green Tea**	1098.34 ± 0.3	158.08 ± 0.5	ND	ND
	**Black Tea**	273.86 ± 0.3	103.04 ± 0.5	ND	ND
	**Turmeric**	49.17 ± 1.6	ND	ND	30.93 ± 0.1

**POOL-3**						
**Analyte Conc. mg/kg of Food Samples**						
**Sample Category**	**Sample Name**	**Isoquercitrin**	**Myricetin**	**Quercetin**	**Chrysin**	**Kaempferide**
**Fruits**	**Apple**	319.33 ± 0.1	199.83 ± 1.9	ND	ND	ND
	**Banana**	ND	68.21 ± 2.7	160.89 ± 1.4	ND	ND
	**Grapefruit**	80.40 ± 1.3	301.74 ± 2.1	ND	32.97 ± 3.7	48.94 ± 0.6
	**Peach**	ND	ND	ND	ND	ND
	**Grapes**	71.82 ± 1.2	ND	ND	ND	ND
	**Plum**	168.43 ± 2.2	42.42 ± 1.4	30.96 ± 3.3	ND	ND

	**Pear**	38.92 ± 2.6	131.19 ± 1.5	ND	ND	ND
**Vegetables**	**Onion**	ND	68.21 ± 1.1	160.89 ± 0.9	ND	ND
	**Cabbage**	43.02 ± 1.8	51.35 ± 2.2	ND	ND	ND
	**Capsicum**	ND	ND	ND	ND	ND
	**Garlic**	ND	80.14 ± 1.0	190.31 ± 1.9	ND	ND
	**Lemon**	96.17 ± 0.1	215.15 ± 2.1	172.25 ± 1.9	ND	ND
	**Tomato**	ND	ND	62.53 ± 1.9	ND	ND
	**Potato**	41.64 ± 1.3	51.95 ± 3.3	ND	ND	ND
	**Spinach**	122.11 ± 1.0	245.75 ± 0.2	70.68 ± 2.0	ND	ND
**Others**	**Corn**	ND	37.46 ± 0.1	ND	ND	ND
	**Rice**	49.14 ± 0.3	ND	ND	ND	ND
	**Kidney beans**	201.10 ± 0.2	ND	ND	112.65 ± 0.2	ND
	**Green Tea**	227.75 ± 0.1	182.15 ± 1.8	72.18 ± 0.1	ND	ND
	**Black Tea**	130.48 ± 0.4	140.87 ± 0.1	54.14 ± 0.7	ND	ND
	**Turmeric**	29.84 ± 0.1	ND	ND	ND	118.31 ± 0.1

**POOL-4**						
**Analyte Conc. mg/kg of Food Samples**						
**Sample Category**	**Sample Name**	**Chlorogenic Acid**	***Trans*-Ferulic Acid**	***Trans*-Resveratrol**	**Herniarin**	**Cinnamic Acid**
**Fruits**	**Apple**	216.39 ± 0.8	81.03 ± 0.3	ND	ND	ND
	**Banana**	73.74 ± 1.5	22.17 ± 3.6	ND	ND	48.38 ± 1.3

	**Grapefruit**	ND	156.79 ± 2.3	80.31 ± 0.4	36.00 ± 1.3	ND
	**Peach**	200.62 ± 0.4	ND	ND	ND	ND
	**Grapes**	34.75 ± 2.5	64.40 ± 1.3	38.24 ± 3.6	ND	23.00 ± 3.1
	**Plum**	54.27 ± 1.4	98.53 ± 1.3	42.69 ± 2.0	ND	35.24 ± 3.6
	**Pear**	99.20 ± 0.2	ND	ND	ND	ND
**Vegetables**	**Onion**	ND	ND	ND	ND	ND
	**Cabbage**	ND	ND	ND	ND	ND
	**Capsicum**	ND	ND	ND	ND	ND
	**Garlic**	ND	ND	ND	ND	ND
	**Lemon**	81.21 ± 0.2	108.62 ± 1.0	49.79 ± 0.8	ND	ND
	**Tomato**	57.51 ± 0.3	28.27 ± 0.2	ND	ND	ND
	**Potato**	153.15 ± 0.5	ND	ND	ND	ND
	**Spinach**	45.63 ± 1.3	142.48 ± 0.7	ND	ND	ND
**Others**	**Corn**	ND	157.01 ± 0.1	BQL	ND	20.79 ± 0.1
	**Rice**	ND	60.07 ± 2.1	ND	ND	ND
	**Kidney beans**	ND	132.78 ± 1.0	BQL	ND	ND
	**Green Tea**	ND	269.20 ± 0.1	54.61 ± 0.9	ND	ND
	**Black Tea**	ND	105.42 ± 0.1	145.10 ± 1.0	ND	ND
	**Turmeric**	ND	67.44 ± 0.7	ND	ND	ND

ND: Not detected
BQL: Below quantification limit

Supplementary Table S4. Tested parameters for the final optimization of HPLC method.

Pool No.	Flow Rate (mL/min)	Injection Volume (µL)	Mobile Phase Gradient
**Pool-1**	0.3	3	10-30% B, 0–1 min; 30-40% B, 1–2 min; 40-80% B, 2–5 min; 80-40% B, 5–7 min; 40-30% B, 7–8 min; 30-10% B, 8–10 min
	0.35	2.5	10-20% B, 0–1 min; 20-40% B, 1–3 min; 40-80% B, 3–6 min; 80-40% B, 6–7 min; 40-20% B, 7–8 min; 20-10% B, 8–10 min
	0.35	2	20-30% B, 0–1 min; 30-40% B, 1–3 min; 40-80% B, 3–5 min; 80-40% B, 5–7 min; 40-30% B, 7–8 min; 30-20% B, 8–10 min
**Pool-2**	0.3	3	10% B, 0–1 min; 10-90% B, 1–7 min; 90% B, 7-8 min; 90-10% B, 8-9 min; 10% B, 9-10 min
	0.3 mL/min, (0-5) min, 0.2 mL/min, (5-10) min, 0.3 mL/min, (10-13) min	2.5	20-30% B, 0–1 min; 30-40% B, 1–4 min; 40-70% B, 4–5 min; 70-80% B, 5–10 min; 80-30% B, 10–11 min; 30-20% B, 11-13 min
	0.35 mL/min, (0-3) min, 0.5 mL/min, (4-5) min, 0.35 mL/min, (5-8) min	2	20-30% B, 0–1 min; 30-40% B, 1–3 min; 40-70% B, 3–4.5 min; 70-40% B, 4.5–6.5 min; 40-30% B, 6.5–7 min; 30-20% B, 7–8 min

**Pool-3**	0.3	3	10-20% B, 0–1 min; 20-70% B, 1–6 min; 70% B, 6–8 min; 70-20% B, 8–9 min; 20-10% B, 9–10 min
	0.35	2.5	20% B, 0–1 min; 20-70% B, 1–6 min; 80% B, 6–8 min; 80-20% B, 8–9 min; 20% B, 9–10 min
	0.35	2	10-20% B, 0–1 min; 20-70% B, 1–5 min; 70% B, 5–7.5 min; 70-20% B, 7.5–8 min; 20-10% B, 8–10 min
**Pool-4**	0.3	3	10% B, 0–1 min; 10-100% B, 1–8 min; 100-10% B, 8–9 min; 10% B, 9–10 min
	0.35	2.5	20% B, 0–1 min; 20-80% B, 1–8 min; 80-20% B, 8–9 min; 20% B, 9–10 min
	0.35	2	10% B, 0–1 min; 10-100% B, 1–6 min; 100-10% B, 6–7 min; 10% B, 7–8 min
